# *PIK3CA* mutations are common in lobular carcinoma *in situ,* but are not a biomarker of progression

**DOI:** 10.1186/s13058-016-0789-y

**Published:** 2017-01-17

**Authors:** Vandna Shah, Salpie Nowinski, Dina Levi, Irek Shinomiya, Narda Kebaier Ep Chaabouni, Cheryl Gillett, Anita Grigoriadis, Trevor A. Graham, Rebecca Roylance, Michael A. Simpson, Sarah E. Pinder, Elinor J. Sawyer

**Affiliations:** 1 Division of Cancer Studies, Guy’s Hospital, King’s College London, London, SE1 9RT UK; 2 Breast Cancer Now Unit, Research Oncology & Cancer Epidemiology, Guy’s Hospital, King’s College London, London, SE1 9RT UK; 3 Evolution and Cancer laboratory, Centre for Tumour Biology, Barts Cancer Institute, Queen Mary University of London, London, UK; 4 Department of Oncology, UCLH Foundation Trust, London, NW1 2PG UK; 5 Medical and Molecular Genetics, Guy’s Hospital, King’s College London, London, UK

**Keywords:** Lobular carcinoma *in situ*, Somatic copy number aberrations, CCND1, PIK3CA, Heterogeneity

## Abstract

**Background:**

Lobular carcinoma *in situ* (LCIS) is a non-invasive breast lesion that is typically found incidentally on biopsy and is often associated with invasive lobular carcinoma (ILC). LCIS is considered by some to be a risk factor for future breast cancer rather than a true precursor lesion. The aim of this study was to identify genetic changes that could be used as biomarkers of progression of LCIS to invasive disease using cases of pure LCIS and comparing their genetic profiles to LCIS which presented contemporaneously with associated ILC, on the hypothesis that the latter represents LCIS that has already progressed.

**Methods:**

Somatic copy number aberrations (SCNAs) were assessed by SNP array in three subgroups: pure LCIS, LCIS associated with ILC and the paired ILC. In addition exome sequencing was performed on seven fresh frozen samples of LCIS associated with ILC, to identify recurrent somatic mutations.

**Results:**

The copy number profiles of pure LCIS and LCIS associated with ILC were almost identical. However, four SCNAs were more frequent in ILC than LCIS associated with ILC, including gain/amplification of *CCND1.* CCND1 protein over-expression assessed by immunohistochemical analysis in a second set of samples from 32 patients with pure LCIS and long-term follow up, was associated with invasive recurrence (*P* = 0.02, Fisher’s exact test). Exome sequencing revealed that *PIK3CA* mutations were as frequent as *CDH1* mutations in LCIS, but were not a useful biomarker of LCIS progression as they were as frequent in pure LCIS as in LCIS associated with ILC. We also observed heterogeneity of *PIK3CA* mutations and evidence of sub-clonal populations in LCIS irrespective of whether they were associated with ILC.

**Conclusions:**

Our data shows that pure LCIS and LCIS co-existing with ILC have very similar SCNA profiles, supporting the hypothesis that LCIS is a true precursor lesion. We have provided evidence that over-expression of CCND1 may identify a subgroup of patients with pure LCIS who are more likely to develop invasive disease, in contrast to *PIK3CA* mutations, which occur too early in lobular tumorigenesis to be informative.

**Electronic supplementary material:**

The online version of this article (doi:10.1186/s13058-016-0789-y) contains supplementary material, which is available to authorized users.

## Background

Lobular carcinoma *in situ* (LCIS) is a non-invasive breast lesion that is typically found incidentally on biopsy but is also often seen in the presence of invasive lobular carcinoma (ILC), which accounts for 10–15% of all invasive breast carcinomas. Hwang et al. showed that ILC and co-existing LCIS share many of the same genetic aberrations [1]. Furthermore, Vos et al. demonstrated the presence of the same truncating mutations in *CDH1* and loss of heterozygosity (LOH) of the wild-type E-cadherin in the LCIS component and adjacent ILC [2].

These studies suggest that LCIS is a non-obligate precursor of ILC in a manner analogous to ductal carcinoma *in situ* (DCIS) preceding invasive ductal carcinoma of no special type (IDC). However, the risk of invasive cancer after LCIS is lower than that with DCIS (2–11 times greater than the risk in the general population, in contrast to DCIS with 20 times greater risk) [3, 4], and the overall rate of progression of pure LCIS to ipsilateral ILC has been shown to be <10% ten years after the diagnosis of LCIS [5, 6]. LCIS is also considered a risk factor for future breast cancer, as not all invasive disease post LCIS presents as ILC and, unlike DCIS, LCIS is also a risk factor for developing invasive cancer in the contralateral breast [7]. Fisher et al. reported that 80% of invasive disease post LCIS is ILC; however, it is likely that the patients in that study did not represent typical cases of classical LCIS (cLCIS) as many were initially diagnosed as having DCIS, but on pathological review were determined to have LCIS [8]. A more recent study has reported much lower rates of ILC post LCIS (27%), although this was still higher than the expected 10–15% [6].

The timescale for the development of invasive carcinoma after an initial diagnosis of LCIS in either breast varies greatly between individuals; one study demonstrated that two thirds of patients developed invasive disease within 15 years; however, another study found that 50% of patients developed ILC up to 15 to 30 years later. This has led some to argue against LCIS as a non-obligate precursor lesion and to suggest that “pure” LCIS may have a different molecular profile compared to LCIS that co-exists with invasive disease, and that the molecular studies cited above have focused on LCIS with associated ILC, rather than pure LCIS.

There are limited studies of pure LCIS but generally these do show similar genetic changes to LCIS associated with ILC, with 16q loss and 1q gain being the most common chromosomal abnormalities. Whilst Mastracci et al. suggested that LOH at 16q was infrequent in 13 cases of pure LCIS [9], comparative genomic hybridization studies on 17 cases of pure LCIS revealed 16q loss in 88% of cases, being the sole detected alteration in 29% [10]. In the latter study, 1q gain was the second most common change, occurring in 41% of tumours and in all cases associated with 16q loss. There is also evidence of E-cadherin loss in both LCIS and atypical hyperplasia with *CDH1* mutations being common in LCIS, but rare in atypical lobular hyperplasia (ALH) [11].

The increased breast biopsy rate associated with screening mammography has led to an increase in the diagnosis of pure LCIS in postmenopausal women [12] with around 3% of needle biopsies identifying pure LCIS [13]. Current guidelines for patients with a diagnosis of LCIS highlight the need for increased surveillance of both the affected and contralateral breasts; however, the optimum management of women with pure LCIS is unclear, as not all women with LCIS will develop invasive disease. Currently in the UK patients with pure LCIS do not receive any further treatment and, even if incompletely excised, no further surgery is performed. There is now convincing evidence from large randomized chemoprevention trials, that 5 years of endocrine therapy reduces the risk of invasive disease after a diagnosis of LCIS by 50%; the NASBP-P1 study demonstrated that 5 years of tamoxifen reduced the development of invasive disease after LCIS from 11% in the control group to 4% in the tamoxifen-treated group [14]. Similarly 5 years of exemestane reduced invasive disease from 13 to 6%, respectively [15]. However, despite this evidence, the use of chemoprevention for LCIS has not become common practice; seemingly clinicians and patients feel that as many cases of LCIS do not progress to invasive disease, the benefits of chemoprevention do not outweigh the potential side effects. It would therefore be invaluable to have biomarkers to predict the likelihood of progression of LCIS, so that appropriate screening and treatment can be offered.

Current biomarker data in LCIS are very limited [16]. There is some evidence to suggest the risk of subsequent invasive disease is associated with high Ki67 expression [17] and that increased expression of hsa-miR-375 contributes to lobular neoplastic progression [18]. Other studies have shown that the five biomarkers known to be important in invasive breast cancer (oestrogen receptor (ER), progesterone receptor (PgR), c-erbB-2, p53 and Ki-67 expression) do not predict progression of LCIS [19]. One of the problems with studies that have tried to identify biomarkers that predict LCIS recurrence is the small number of cases analysed due to the rarity of pure LCIS.

The aim of the present study was to identify genetic changes that could be used as biomarkers of progression of LCIS to invasive disease. Ideally this should be done in a cohort of patients with pure LCIS, who have progressed to invasive disease, compared to a cohort that have not, but such patients are very rare. We therefore chose to do our discovery phase using patients with pure LCIS and comparing their genetic profiles to patients with LCIS who presented contemporaneously with associated ILC, based on the hypothesis that the latter represents LCIS that has already progressed.

## Methods

### Samples

Archival cases were identified from the study to investigate the genetics of lobular carcinoma *in situ* in Europe (GLACIER) (06/Q1702/64). In this study blood samples and formalin-fixed paraffin-embedded (FFPE) tumour samples were available for women with LCIS from 97 hospitals throughout the UK, together with data on known hormonal risk factors for breast cancer. For the discovery phase 30 patients with classic pure LCIS (pure-cLCIS) and 30 with classic LCIS associated with ILC (inv-cLCIS) were selected for SNP-array analysis to assess copy number changes, together with 7 patients with classic LCIS with ILC who had fresh-frozen lesions stored in the KHP Cancer Biobank (NHS REC ref. 12-EE-0493), which were used for whole exome sequencing in order to assess the frequency of mutations in inv-cLCIS.

Following independent review by a breast histopathologist (SEP) to confirm the diagnosis, samples were visually assessed to determine the presence of sufficient malignant tissue for needle dissection under a light microscope (macrodissection). E-cadherin expression was also assessed by immunohistochemical analysis and in all cases E-cadherin staining was absent within the LCIS/ILC with the exception of one pure LCIS sample, which had occasional foci of patchy weak membrane staining.

Up to 20 10-μm sections were stained using Nuclear Fast Red (Sigma) and macrodissected to separate the LCIS and invasive components. In order to obtain enough DNA to perform these experiments, all the pure-cLCIS foci in a single FFPE block were macrodissected (maximum area 2 cm^2^). In samples from 23 women with inv-cLCIS, this was on the same block as the invasive tumour and in samples from 5 women it was on an adjacent block. Tumour DNA was extracted using the QIAamp DNA FFPE Tissue Kit (Qiagen) for archival specimens and the DNeasy Blood & Tissue Kit (Qiagen) for fresh-frozen samples. Patient-matched germline DNA was extracted from peripheral blood samples using the Nucleon product chemistry (Tepnel, Manchester, UK). DNA was quantified using Quant-iT™ PicoGreen® dsDNA Assay Kit (Life Technologies).

For the validation of potential biomarkers, we studied samples from 37 patients with pure LCIS, who were diagnosed between 1980 and 2011 and had been followed up for longer than 6 months (median follow up was 81 months, range 34–333). A table summarizing all the clinico-pathological data on the samples used in this study can be found in Additional file 1: Table S1 (discovery set) and Additional file 1: Table S2 (validation set).

### SNP arrays

Samples were hybridised onto the Oncoscan™ Affymetrix array. The array uses molecular inversion probe (MIP) technology to detect 335,000 markers for genome-wide, allele-specific copy numbers with enhanced coverage of known cancer genes, and which also provides a mutation score for the likelihood of the sample containing a somatic mutation in a series of key cancer genes (http://www.affymetrix.com). A minimum of 200 ng DNA for each sample was used. Labelling and hybridization were outsourced to Affymetrix (Santa Clara, CA, USA).

Raw array data was preprocessed with Nexus 7.5 software (BioDiscovery), applying the SNPRank segmentation algorithm with a minimum of 10 probes per segment. Tumour Aberration Prediction Suite (TAPS) 2.0 [20] was used to determine absolute copy number and for categorisation into: gains (copy number (CN) =/> 3); amplification (CN>/=5), losses (CN =/< 1); and copy neutral LOH (cnLoH). Samples found to have whole genome duplication were corrected to a diploid state. Frequency plots were made using the copy number package in R 3.0.0 (https://www.bioconductor.org/packages/release/bioc/html/copynumber.html).

Frequency tables of the resulting somatic copy number aberrations (SCNAs) were generated for pure LCIS, the LCIS associated with ILC, and the paired ILC, and the prevalence of SCNAs were compared using Fisher’s exact test. Genomic regions with statistically significant differences (*P* < 0.05) but reported in regions of known HAPMAP copy number variations (CNVs) (http://hapmap.ncbi.nlm.nih.gov/) were removed.

Two-way hierarchical clustering of the pure LCIS, inv-LCIS, and ILC samples was performed using Manhattan distance and Ward’s clustering method in the NMF package in R (http://nmf.r-forge.r-project.org/aheatmap.html) based on type SCNA (=/>100 probes) occurring on each p and q arm. The raw data are available (https://www.ncbi.nlm.nih.gov/geo/query/acc.cgi?acc=GSE88909).

### Immunohistochemical analysis (IHC) and fluorescence *in situ* hybridization (FISH)

*CCND1* FISH was performed using 4-μm tissue sections treated with SPOT-Light® Tissue Pretreatment kit (Life Technologies) prior to hybridisation with Abbott Molecular Vysis LSI *CCND1* and chromosome 11 enumeration probe kit (Abbott Molecular Inc.) according to the manufacturer’s instructions. The 11q13 probe covers a genomic region of 378 kb and includes the following genes: *CCND1*, *FGF19* and *FGF4. CCND1* and chromosome 11 enumeration probe (CEP11) foci were counted in a minimum of 20 cells and an average was determined. The ratio of *CCND1* to CEP11 foci was calculated and gain was defined as a ratio of 1.5–2.5 and amplification >2.5.

*CCND1* IHC was performed on 3-μM FFPE tissue sections using clone SP4-R (Ventana Medical Systems Inc.) on an automated staining system (VENTANA BenchMark ULTRA, Roche). *CCND1* was scored as described previously by Reis-Filho et al. [21] (equivalent to the Allred scoring system for ER) and based on the proportion of stained nuclei (scored 0–5: 0 = none; 1 = <1/100; 2 = 1/100–1/10; 3 = 1/10–1/3; 4 = 1/3–2/3 and 5 = >2/3) and intensity (0–3: 0 = no staining; 1 = weak staining visible only at high magnification; 2 = moderate staining and 3 = strong staining visible at low magnification). The two scores were combined to give a total score (0–8: 0–2 = low; 3–5 = intermediate and 6–8 = high).

### Whole exome sequencing (WES)

Libraries were prepared from tumour and paired germline DNA using the SureSelect Human All Exon 50 Mb kit (Agilent) and sequenced on Illumina HiSeq 2000 to a mean depth > ×100. Subsequent analysis was performed using our in-house pipeline; in brief, sequencing reads were aligned to the reference human genome hg19 using NovoAlign (http://www.novocraft.com/products/novoalign/), Samtools [22] was used to create a pileup file and VarScan2 [23] was used to call somatic mutation and indels, annotated using ANNOVAR [24] and cross-referenced with dbSNP and 1000 Genomes. Somatic mutations were called if there was a minimum of × 30 coverage and the mutation was present in at least 10% of reads.

### Sanger sequencing

Using standard protocols on the Applied Biosystems 3730xl DNA analyzer with the Finch TV software (Geospiza), Sanger sequencing confirmed the presence of mutations detected by the Oncoscan array or exome sequencing. A restriction enzyme enrichment protocol on stock DNA was used [25] for mutations in exon 9 of PIK3CA (c1624G_A and c1633G_A).

### Assessing tumour heterogeneity using the SNP-array

For regions with a copy number score between 1 and 2 on TAPs analysis, i.e. not being designated as loss by the algorthim but showing some loss visually, we used highly polymorphic microsatellite markers to assess whether these segments represented areas of sub-clonal loss or were just experimental variation in calling of copy number 2, by extracting DNA from different areas of LCIS microdissected using the PALM MicroBeam laser capture microscope (Carl Zeiss). Microsatellite markers on 16q (D16S752) or 6q (D6S1627) were used as a “control”, as these regions were designated as loss (copy number 1) by TAPs.

Having confirmed that the segments that did not reach the threshold for loss did represent subclonal events, we plotted the transformed log2 ratio and transformed b-allele frequency (allelic imbalance score from TAPS 2.0 output) for each segment of loss to count the number of these segments that were subclonal per sample. Samples in which the three clusters (absolute copy numbers 1, 2 and 3) could not be defined due to poor quality of the DNA or poor hybridisation were excluded (three cILC, three inv-cLCIS and 5 pure cLCIS samples). The segment with an absolute copy number of 1 with the lowest log2 ratio and highest allelic imbalance score in a sample was assumed to be a clonal event and to be present in 100% of the tumour cells (generally 16q), and it was used as the reference for copy number 1. The Euclidian distance was calculated from the centroid of all the diploid segments to this reference segment. Next the distance of the remaining segments from the diploid centroid was calculated as a proportion of this reference distance (Additional file 2: Figure S1). A sample-specific threshold was established by examining the distribution of the proportions of the segments considered to be diploid by TAPS output. The threshold for sub-clonality was defined as being outside the 99^th^ percentile of this distribution. Applying this threshold to all the segments considered to be copy number 1 in the TAPS output identified sub-clonal segments.

## Results

### SNP array-based copy number analysis of classical LCIS

**Fig. 1:**
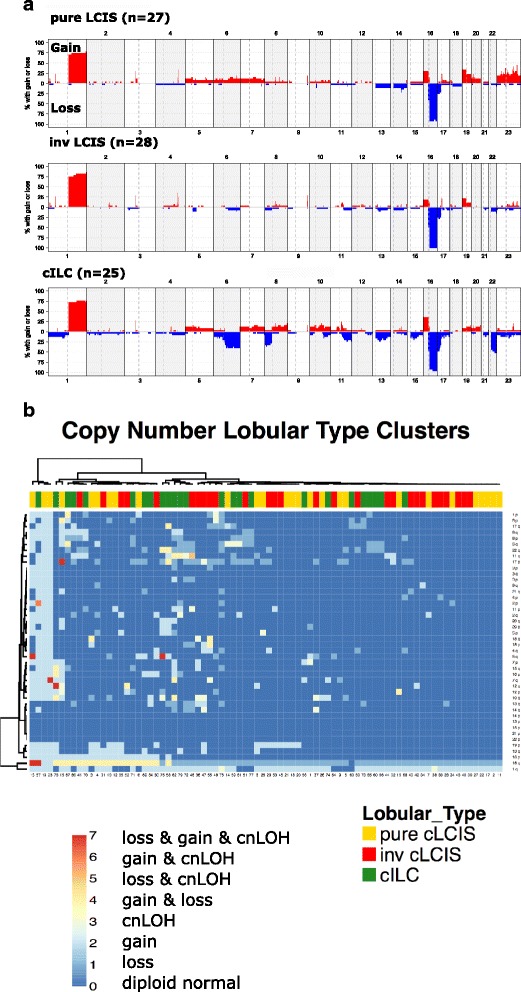
**a** Summary of copy number changes in pure classic lobular carcinoma *in situ* (*pure-cLCIS*), classic lobular carcinoma *in situ* associated with invasive lobular cancer (*inv-cLCIS*) and classic lobular invasive cancer (*cILC*). **b** Two-way hierarchical clustering of the pure-cLCIS, inv-cLCIS and cILC based on type of somatic copy number aberration (SCNA) (=/>100 probes) occurring on each p and q arm. (*cnLOH* = copy neutral loss of heterozygosity)

In the discovery set, adequate DNA for hybridization onto the Oncoscan V2 array was extracted from 27/30 samples of pure classic LCIS, and from the 30 samples of classic LCIS associated with ILC, 28 had adequate DNA extracted from the LCIS component and 25 from the ILC component. Frequency plots of gains and losses within each group revealed similar patterns, Fig. 1a.

Pure-cLCIS and inv-cLCIS had remarkably similar SCNAs. There was no evidence that pure-cLCIS was any less genetically re-arranged than inv-cLCIS, with pure LCIS samples not clustering together but scattered throughout the inv-LCIS and ILC samples on hierarchical clustering (Fig. 1b). In fact aneuploidy was more common in the pure-cLCIS group with three cases being triploid, and one case having complex genetic re-arrangements suggestive of chromothripsis; none of the inv-cLCIS cases had evidence of such changes (Additional file 2: Figure S2).

All cLCIS samples, irrespective of whether or not they were associated with ILC, had loss or cnLOH of 16q as did 24/25 of the paired cILC. One ILC sample had visual evidence of low-level 16q loss but did not reach the threshold for copy number 1 using the TAPS algorithm, most likely due to normal tissue contamination, as there was no evidence of E-cadherin expression on IHC. As expected, gain or cnLOH of 1q was the second most frequent change occurring in 21/27 pure c-LCIS samples (78%), 25/28 inv-cLCIS samples (89%) and 21/25 cILC samples (84%). These two genetic changes clustered strongly together (Fig. 1b), and were the sole SCNAs in five pure - cLCIS and 3 inv-cLCIS samples. Other frequent SCNAs that occurred in all subtypes were gain of 16p (8/27 pure-cLCIS, 5/28 inv-cLCIS and 9/25 cILC samples) and gain of 19p (9/27 pure-cLCIS, 6/28 inv-cLCIS and 2/25 cILC samples) and loss of 17p (7/27 pure-cLCIS, 8/28 inv-cLCIS and 12/25 cILC samples). Smaller regions (<10 Mb) of gain were found on 1p34.1, 3p14.1, 9q33.3 and 17q11.2, and of loss were found on 17p13.1 and these occurred with similar frequency in both pure-cLCIS and inv-cLCIS (Additional file 1: Table S3). Of these, only 3p14.1 gain, containing the transcription factor *MITF*, and loss on 17p13.1 loss (*TP53*), were found with similar or increased frequency in the paired ILC.

Amplifications (defined as copy number =/> 5) were found in 15 pure-cLCIS samples and 14 inv-cLCIS samples (Additional file 1: Table S4). The most common amplifications in cLCIS were on 1q, encompassing *AKT3* (4/27 pure-cLCIS, 4/28 inv-cLCIS and 4/25 ILC samples) and 11q13, encompassing *CCND1* (0/27 pure-cLCIS, 2/28 inv-cLCIS and 5/25 cILC samples).

The only SCNAs that were significantly different between pure-cLCIS and inv-cLCIS were five small regions of gain, more common in pure LCIS, three of which contained a single gene: Xp11 (*KLF8*), 20p12.1 (*MACROD2*) and 17q11.2 (*RAB11FIP4*) (Table 1).

**Table 1:** Summary of somatic copy number aberrations showing a difference in frequency between pure-cLCIS, inv-cLCIS and cILC

Chr	Region	Genes	Type of SCNA	PURE c-LCIS (27)	INV-cLCIS (28)	cILC (25)	*P*_values		
							Pure cLCIS vs INV-cLCIS	INV-cLCIS vs cILC	Across all 3 groups
5	39436375-39620648 (p13.1)	LOC101926940	Gain	4	0	3	0.05	0.1	0.1
5	129741359-131422972 (q23.3 – q31.1)	HINT1, LYRM7, CDC42SE2, RAPGEF6, FNIP1,MEIKIN, ACSL6, IL3, CSF2	Gain	4	0	2	0.05	0.2	0.09
6	82391438-171115067 (q14.1 – q27)	Many including: MAP3K7, FOXO3, ESR1, IGF2R, MAP3K4, etc.	Loss	0	2	10	0.5	0.007	0.00007
8	5412833-8927086 (p23.2-p23.1)	MCPH1, ANGPT2, AGPAT5, XKR5, Defensins, FAM66B,SPAG11B &A,CLDN23, ERI1	Loss	1	2	9	1	0.04	0.002
10	75541103-76515425 (q22.2)	CHCHD1,VCL,PLAU,ADK,AP3M1, CAMK2G, NDST2	Gain	2	0	5	0.2	0.05	0.03
11	68961001-71551048 (q13.3 – q13.4)	CCND1, MYEOV, FGF4, FGF3	Gain (Amp)	0	4 (2)	6 (5)	0.1	0.1	0.02
17	29779560-29899917 (q11.2)	RAB11FIP4	Gain (Amp)	5 (1)	0	0	0.02	0.005	0.005
18	727180-742194 (p11.32)	YES1	Gain (Amp)	7 (1)	2	0	0.07	0.02	0.02
X	55670623-57693679 (p11.21)	KLF8	Gain	10	0	1	0.0003	0.00004	0.00004
20	14695735-15225214 (p12.1)	MACROD2	Gain	6	0	1	0.01	0.006	0.006
22	47751337-51304566 (q13.31 - q13.3)	BRD1, HDAC10, MAPK12. MAPK11 ….	Loss	2	4	13	0.6	0.005	0.0003

*Pure-cLCIS* pure classic lobular carcinoma *in situ*, *inv-cLCIS* classic lobular carcinoma *in situ* associated with invasive lobular cancer, *cILC* classic invasive lobular cancer, *Chr* chromosome, SCNA somatic copy number aberration

Only one inv-cLCIS/cILC pair clustered together (Fig. 1b); however, this was because in the majority of inv-cLCIS samples the invasive component had the same SCNAs as its paired LCIS but had also acquired additional genetic changes, with SCNAs being more common in cILC than in inv-cLCIS (*P* = 0.003) (Additional file 2: Figure S3). Four regions had an increase in frequency from pure-cLCIS through to inv-cLCIS and cILC: loss of 6q, 8p23 and 22q13 and gain/amplification of 11q13 (Table 1). Among the three regions of loss the most significant increase in frequency came in the transition from inv-cLCIS to cILC.

**Fig. 2:**
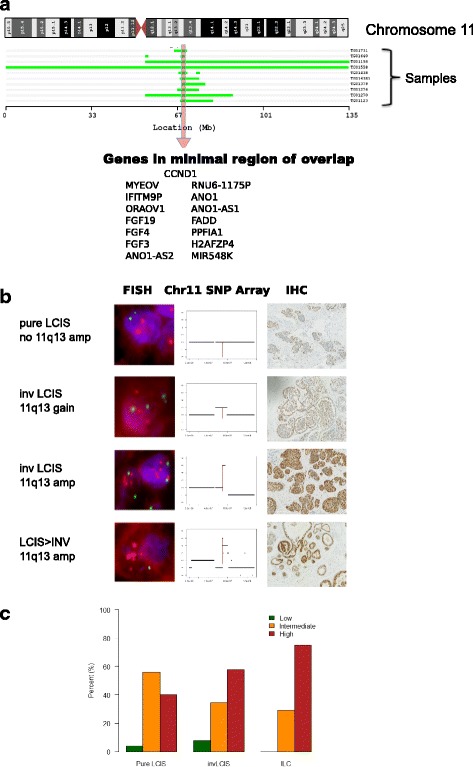
**a** Minimal region of gain/amplification on 11q; **b** correlation of copy number change at 11q13 with fluorescence *in situ* hybridization (*FISH*) using the probe for *CCND1* and Cyclin D1 immunohistochemical analysis (*IHC*); **c** frequency of Cyclin D1 expression as measured by IHC in the discovery set. (*SNP* = single nucleotide polymorphism, *LCIS* = lobular carcinoma in situ, *Inv LCIS* = lobular carcinoma *in situ* associated with invasive lobular cancer)

Gain/amplification of 11q13, was considered potentially the most useful biomarker of LCIS progression, as none of the pure LCIS samples had gain/amplification, but four (two gain, two amplification) of the inv-cLCIS did, with amplification becoming more common in the invasive component. Analysis of the amplicons in our samples revealed that the minimal region of gain/amplification was Chr11: 68961001–70183017, Fig. 2a. This region includes a number of potential oncogenes including *CCND1*, *FGF19*, *FGF4* and *FGF3.* FISH using a probe encompassing *CCND1*, *FGF19* and *FGF4* confirmed the presence of gain/amplification of 11q13 in all samples with gain/amplification on the SNP array, Fig. 2b. IHC was performed on all samples in the discovery set and this showed that all samples with gain/amplification of 11q13 also had high expression (score ≥6) of cyclin D1. IHC also detected other samples with high protein expression but no evidence of amplification. There was a trend towards higher cyclin D1 expression from pure LCIS through to inv-cLCIS and ILC (*P* = 0.07) (Fig. 2c).

In order to assess whether expression of cyclin D1 could be a potential marker of progression, IHC was performed on a different set of samples from 32 patients from the GLACIER study, who had long term follow up and FFPE blocks available, and had not received chemoprevention or had undergone mastectomy (Additional file 1: Table S2). Of these 32 patients, 8 had developed invasive disease with a median time to recurrence of 69 months (range 34–175). Four had developed ipsilateral recurrence (two ILC, one IDC, one ILC and IDC) and four, contralateral invasive disease (one tubular carcinoma, one IDC and two ILC) (Additional file 1: Table S2a). The latter were excluded from the analysis as they do not represent direct clonal progression of LCIS. Cyclin D1 protein overexpression was associated with recurrence (*P* = 0.02, Fisher’s exact test) with high expression in 4/4 samples from patients with pure LCIS that progressed to ipsilateral invasive disease, compared to only 8/24 samples from patients with pure LCIS, with no evidence of recurrence after a minimum of 60 month follow up.

IHC had a positive predictive value (PPV) of 33% and negative predictive value (NPV) of 100%. FISH was only performed on samples in the validation set with high cyclin D1 expression on IHC, as our data and others [26] have shown correlation between amplification and high expression on IHC. One of the four patients that developed ipsilateral invasive disease (Fig. 2c-iv) had amplification of *CCND1* and subsequently developed ILC, which was treated with mastectomy and endocrine therapy but recurred 2 years later in the mastectomy scar. The invasive recurrence also had *CCND1* amplification. One of the 24 patients with long-term follow up and no evidence of recurrence had evidence of CCND1 gain (*P* = 0.27, Fisher’s exact test).

### Mutation analysis

Seven fresh-frozen samples of classic ILC with associated LCIS were selected for whole exome sequencing (WES) and the two components were macrodissected under the light microscope. Adequate DNA for WES of both the ILC and LCIS components was obtained from only one patient. In the remaining samples three had adequate DNA from the LCIS component and three from the ILC.

In the single paired LCIS-ILC sample, 13 mutations were shared between the two components, including two *PIK3CA* mutations and one truncating *CDH1* mutation, a frameshift mutation in *COX15* and stop-gain in *DOCK2* (Additional file 1: Table S5a). There was no evidence that these mutated genes targeted a particular biological pathway (http://geneontology.org/). The ILC component had 23 mutations not found in the LCIS component, of which 5 were transcription factors (*RB1, ARID4A, VGLL3*, *ZNF341* and *SIX1)* (Additional file 1: Table S5b). Similarly the LCIS component had mutations in 10 genes, not found in the ILC component, suggesting there are also driver and passenger mutations at the pre-invasive stage (Additional file 1: Table S5c).

**Fig. 3:**
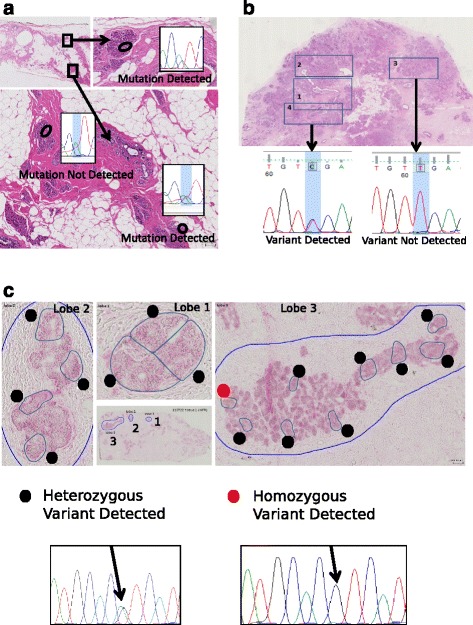
**a** Heterogeneity of *PIK3CA* mutations in two pure classic lobular carcinoma *in situ* (pure-cLCIS) samples; **b** heterogeneity of *ERBB2* mutation within the LCIS component but not in invasive lobular carcinoma (ILC); **c** heterozygous and homozygous *PIK3CA* mutations in invasive cLCIS associated with ILC (inv-cLCIS)

Analysis of the pooled exome sequencing data from all samples revealed that *CDH1* and *PIK3CA* were the most common somatic mutations*,* which co-existed in 3/4 LCIS and 3/4 ILC samples (Table 2). The finding that *PIK3CA* mutations were as common as *CDH1* mutations in LCIS was surprising and we therefore sequenced the commonest mutations in exon 9 and 20 of *PIK3CA* (c3140A > G, c3140A > T, c1624G > A, c1633G > A, c1258T > C, c1636C > A) in the same samples that underwent Oncoscan array analysis (27 pure-cLCIS and 28 inv-cLCIS samples) to assess their frequency and determine whether *PIK3CA* mutations could be used as a biomarker for LCIS progression. Due to the limited amount of DNA, the Oncoscan MIP array *PIK3CA* mutation score was used as a guide as to which mutations to assess in each sample. All *PIK3CA* mutations with an Oncoscan score >4.5 were sequenced using Sanger sequencing. Sanger sequencing DNA extracted from macrodissected samples did not detect any mutations; however, the use of restriction enzymes or microdissection of small areas of tissue using the laser capture microscope (LCM) to minimise normal tissue contamination, confirmed those mutations (Fig. 3).

**Table 2:** Whole exome sequencing of four invasive lobular cancer (ILC) samples and four lobular carcinoma *in situ* (LCIS) samples (one paired): mutations occurring in two or more samples

Gene	Chrom	Number of ILC samples with mutation	Number of LCIS samples with mutation	Total
HSPG2	1	1	1	2
ROR1	1	1	1	2
SMG7	1	1	1	2
TSSC1	2	2	0	2
PIK3CA	3	3	3	6
DOCK2	5	1	1	2
HAND1	5	2	0	2
UTP23	8	2	0	2
CACNB2	10	1	1	2
COX15	10	1	1	2
OR56B1	11	1	1	2
DDX11	12	1	1	2
PROSER1	13	1	1	2
TPTE2	13	1	1	2
PAK6	15	2	0	2
CDH1	16	3	3	6
PTRF	17	1	1	2
ATP11C	X	1	1	2
ATRX	X	1	1	2

In classic cases, 5/27 pure-cLCIS samples (18.5%) and 6/28 inv-cLCIS samples (21.5%) had *PIK3CA* mutations. In the latter group all of the mutations present in the LCIS were also present in the paired ILC, with the exception of two samples. One contained three different *PIK3CA* mutations in the LCIS component and only two were transferred to the paired ILC, and the other did not have enough DNA to test the ILC component (Table 3).

**Table 3:** Frequency of PIK3CA mutations in classic lobular carcinoma *in situ* (LCIS)/invasive lobular cancer (ILC)

Mutation (total number of cases)	Pure LCIS (27)	inv-LCIS (28)	ILC (25)
PIK3CA_pH1047R_c3140A_G	3 (1558,1717, 04078^a^)	3 (1731, 1965, *1604*)	2^a^ (1731,*1604*) 1965- no tissue left
PIK3CA_pH1047R_c3140A_T	0	0	0
PIK3CA_pE542K_c1624G_A	1 (1339)	3 (1063,*1604,* 1640)	3 (1063, *1604,* 1640)
PIK3CA_pE545K_c1636C_A	0	0	0
PIK3CA_pE545K_c1633G_A	1 (1078)	2 (1270, *1604*)	2 (1270,1126)
Total number of cases with PIK3CA Mutations	5	6	6

^a^sample IDs (*italics represent samples with multiple PIK3CA mutations)*

These findings suggest that like *CDH1*, *PIK3CA* mutations might be early events in lobular tumourigenesis, but are not a potential biomarker for progression from LCIS to ILC.

### Assessing tumour heterogeneity using Oncoscan MIP array data

Exome sequencing revealed that mutations occurred in 10–24% of the reads in the LCIS samples and in 10–40% of the ILC samples. As these samples were macrodissected it is highly likely that there was significant normal tissue contamination, particularly in the ILC samples due to the characteristic single cell file infiltration of breast stroma seen in this subtype of carcinomas. However it is unlikely that there was 80% normal tissue contamination in the LCIS samples and we therefore tested the hypothesis that heterogeneity within the LCIS could also be contributing to the low number of reads with mutations. We sequenced mutations from multiple areas within selected samples microdissected by LCM (two to six areas). In two pure-cLCIS samples there was evidence of heterogeneity of the *PIK3CA* mutation, Fig. 3a. In a sample with an *ERBB2* mutation there was evidence of heterogeneity within the *in situ* component but not in ILC (Fig. 3b), and in one inv-cLCIS sample there was evidence of heterozygous and homozygous *PIK3CA* mutations (Fig. 3c).

**Fig. 4:**
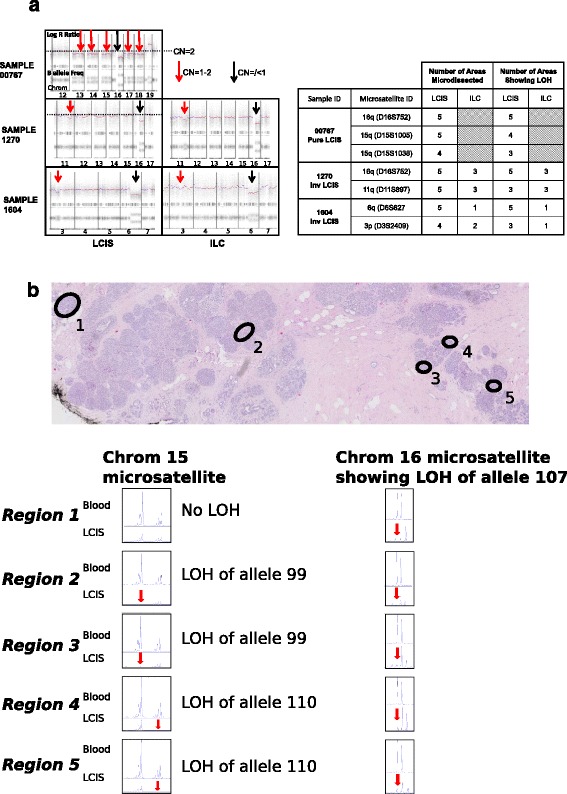
**a** Sub-clonal segments on copy number analysis; **b** microsatellite markers confirm that sub-clonal segments on copy number analysis show heterogeneous loss. (*LOH* = loss of heterozygosity)

We found further evidence of genetic heterogeneity within LCIS when analysing the SNP-array data. Although the majority of the SCNAs reached the threshold for loss (copy number 1) we also identified segments that did not reach these thresholds (Fig. 4a). In order to ascertain whether these regions could represent sub-clones we used highly polymorphic microsatellite markers for each of the heterogeneous regions (D15S1005 and D15S1038, D11S897, D3S2409) and performed standard LOH analysis on DNA extracted from different areas of microdissected LCIS. Microsatellite markers on 16q (D16S752) or 6q (D6S1627) were used as the control, as these regions reached the threshold for loss.

The microsatellite analysis confirmed that where SCNAs did not reach the threshold for loss in LCIS there was evidence of heterogeneity, with LOH occurring in 60–80% of the microdissected regions (Fig. 4b). Following confirmation that these regions did represent sub-clones, we calculated the frequency of such segments in the different subsets of lobular cancer using the SNP-array data as described in “Methods”. There was a non-significant trend for the number of samples with sub-clones to increase from pure LCIS to ILC (*P* = 0.08) in the classic form of the disease.

## Discussion

Our study confirms the finding of previous studies that LCIS has the same molecular changes as co-existing ILC. We have also shown that pure LCIS and LCIS associated with ILC (inv-LCIS) have very similar SCNAs, supporting the hypothesis that pure LCIS is a precursor lesion. Only five regions were significantly different between pure-cLCIS and inv-cLCIS and these were all more common in pure LCIS, so did not represent markers of progression. Three of the regions were small, containing just one gene: chr17 - RAB11FIP4; chr20 - MACROD2, overexpression of this gene has been implicated in oestrogen-independent growth [27]; and chrX - KLF8, oncogenic in ovarian but not breast cancer cell lines [28]. There was also a similar region on chr18, that was more common in both pure and inv-cLCIS compared to ILC, containing YES1, a SRC proto-oncogene that has recently been identified as a possible therapeutic target in basal breast cancer [29]. It is possible that these regions contain genes that hinder development of the invasive phenotype. A similar finding was made in DCIS (albeit with different genomic regions) and the authors hypothesized that these regions could contain genes that provide a selective advantage under local conditions but also inhibit invasion [30].

We identified four SCNAs that increased in frequency from pure cLCIS to inv-cLCIS and finally ILC: loss of 6q14.1-27, 8p23.2-23.1, and 22q13.31-13.33 and gain of 11q13.3. For the three regions of loss the most significant increase in frequency came in the transition from inv-cLCIS to cILC. Both 6q and 8p23 loss have also been found to be more common in invasive ductal carcinoma (IDC) than in paired DCIS [30] suggesting that there may be tumour suppressor genes in these chromosomal regions important in the transition of *in situ* to invasive carcinoma in both DCIS and LCIS. A recent study integrating SCNAs, promoter methylation and gene expression profiles in luminal B breast cancers showed that 88% of the potential tumour suppressor genes were located on 6q [31]. Deletions of 8p have been described in 50% of IDC and 37% of lobular cancers [32] and the minimal region of loss in our study (8p23.2-23.1) includes *MCPH1*, a potential tumour suppressor gene [33, 34] encoding a DNA damage response protein which has also been implicated in breast cancer predisposition [35]. Similarly, loss of 22q13 has been described in IDC (not present in paired DCIS) and ILC (but also present in paired LCIS) [36, 37].

Gain/amplification of 11q13.3 was considered the most useful SCNA to take forward as a potential practical biomarker of LCIS progression as unlike the regions of loss, the main increase in frequency occurred between the pure-cLCIS and inv-cLCIS sub-groups. There was also evidence of increasing amplitude of copy number gain between the paired inv-cLCIS and ILC samples. The region contains *CCND1* and other potential oncogenes such as *FGF3, FGF4* and *MYEOV* and there is good evidence that this region is relevant to breast cancer [38]. Two large studies of DCIS (approximately 400 patients) have reported amplification of *CCND1* in 10–12.6% of patients with pure DCIS and 14.8–17.4% of patients with DCIS associated with invasive breast cancer, with the majority of patients having amplification in the paired invasive component [39, 40]. In a much smaller series of 20 patients with florid LCIS (a subtype putatively more likely to associated with invasive disease) 25% had *CCND1* amplification [41]. This, together with the finding that *CCND1* amplification is usually homogeneous within breast carcinomas, suggests that it is an early event in the development of some breast cancers [42].

Other studies have identified correlation between over-expression of cyclin D1 and amplification of 11q13 and this over-expression has been associated with an increased risk of recurrence in ILC [43]. One early study of ILC suggested that cyclin D1 protein overexpression may play a role in the transition of LCIS to ILC, as the majority of ILC samples had over-expression but with little evidence in surrounding LCIS, although the number of patients with LCIS was not stated.

Our data support their finding that cyclin D1 over-expression is important in the transition of LCIS to ILC, but we have also shown that some cases of LCIS also over-express cyclin D1 and this may be a marker of progression to ILC [44]. In a genome-driven classification of over 7500 breast tumours, amplification of 11q13.3 was associated with a sub-group of ER-positive breast tumours with a poor prognosis and chemo-resistance. This sub-group accounted for only 3.1% of tumours in this series of mainly IDC [45]. However, in our study 24% of cILC had 11q13.3 amplification in keeping with other series showing 11q13.3 amplification is more frequent in ILC than IDC [46]. In our validation set, albeit very small, we found that cyclin D1 over-expression may identify a subset of pure LCIS that is likely to progress to ILC. As around 40% of ILC have high expression of cyclin D1 [43], this represents a significant subset of ILC.

Exome sequencing of this small number of LCIS and ILC did not identify any potential biomarkers of LCIS progression. It did, however, show that activating *PIK3CA* mutations are as common in LCIS as *CDH1* mutations. Activating *PIK3CA* mutations are well-described in both ILC and IDC, occurring in 48% of ILC (as the second most common mutations after *CDH1*) and 33% of IDC [47, 48]. *PIK3CA* mutations have previously been reported by Christgen et al. [49] in 1/3 patients with LCIS associated with ILC and by Sakr et al. [48] in 7/19 patients. We confirmed the frequency of *PIK3CA* mutations in a larger set of LCIS by Sanger sequencing, which revealed no difference in the frequency of *PIK3CA* mutations in inv-cLCIS compared to pure-cLCIS. The only other study to assess *PIK3CA* mutations in pure LCIS was by Sakr et al. who found no evidence of *PIK3CA* mutations in three patients with pure LCIS after targeted sequencing [48]. The frequency of *PIK3CA* mutations in ILC in our study is lower than that reported by The Cancer Genome Atlas (TCGA) and Sakr et al.; however, this is likely to be because both those studies used next-generation sequencing to assess the whole gene, whereas we targeted only the common mutations.

So, although they are not a useful biomarker of LCIS progression, *PIK3CA* mutations are an early event in lobular tumorigenesis leading to abnormal proliferation of the breast epithelium, but importantly, they do not appear to be the critical event leading to invasive malignancy. This is supported by the findings of Ang et al. who identified frequent *PIK3CA* mutations in non-invasive proliferative breast lesions including DCIS, inv-LCIS and one case of pure LCIS [50, 51].

A comprehensive analysis of 127 ILC by the TCGA has shown that *CDH1, PIK3CA, TBX3, FOXA1* and *RUNX1* are the most commonly mutated genes [47]. We found no evidence of *TBX3*, *FOXA1* or *RUNX1* mutations in the four LCIS samples on which we performed exome sequencing, and identified only one *TBX3* mutation in the four ILC samples. The only other known driver gene mutated in LCIS was *ATRX*, frequently mutated in neuroblastoma, low-grade glioma and glioblastoma but not common in breast cancer. In the present series we report splice site mutation in two additional well-known drivers of cancer, *MAP2K4 and RB1* in ILC, but not LCIS,

The TCGA data also showed that AKT signalling is strongly activated in ILC and homozygous losses of the *PTEN* locus (10q23) occurred in 6% of ILC [47]. We found no evidence of homozygous deletions of 10q23 in LCIS in our samples. One case of ILC did have a homozygous deletion at the PTEN locus, whilst this was not evident in the paired LCIS, suggesting it is a later event in lobular tumourigenesis. Interestingly the region encompassing *AKT3* was the locus most frequently amplified in both pure cLCIS and inv-cLCIS and paired cILC. So, although *PIK3CA* mutations do not appear to be the trigger for malignant transformation in lobular cancer, it is possible that progression of LCIS may be related to acquisition of mutations or alterations in other components of the PI3K/Akt pathway; for example, expression profiling studies have shown that PIK3R1 is significantly downregulated in the stepwise progression from normal epithelium to LCIS to ILC [52].

With the advent of next-generation sequencing intra-tumoural heterogeneity has been found to be widespread in invasive cancer; however, there are few data on the intra-tumoural heterogeneity of *in situ* breast cancers. We developed a relatively crude method to assess heterogeneity using SNP arrays and this clearly showed that sub-clonal SCNAs increase in frequency from *in situ* to invasive lobular carcinoma. It remains to be seen whether a more sensitive measure of clonal diversity could be used as a biomarker of progression to invasive disease, as it is in Barrett’s oesophagus [53, 54]. Of interest, we have also shown evidence of passenger mutations in LCIS not transmitted to the invasive component, suggesting that, like invasive disease, there is an early sub-clone expansion process [55], with at least one acquiring critical mutations and developing into invasive disease. Driver mutations that are sub-clonal in the pre-invasive state then become clonal in the invasive stage.

## Conclusions

In conclusion our data have shown that pure LCIS and LCIS co-existing with ILC have very similar SCNA profiles, suggesting that pure LCIS is not intrinsically a different process that is less likely to develop into invasive disease. We have identified four SCNAs that are important in the transformation of LCIS to ILC and provided evidence that over-expression of cyclin D1 may identify a subgroup of LCIS more likely to develop invasive disease. This needs confirming in larger studies, although this will be challenging, as there are few series with long-term follow up of pure LCIS. We have also shown that *PIK3CA* mutations are common in LCIS and that there is genetic heterogeneity within LCIS, just as in ILC.

## Additional files

Additional file 1: Table S1a. Clinico-pathological features of the discovery set - pure LCIS. **Table S1b.** Clinico-pathological features of the discovery set - inv-LCIS and ILC. **Table S2a.** Validation set: characteristics of the eight pure LCIS tumours that recurred. **Table S2b.** Validation set: characteristics of pure LCIS tumours that did not recur. **Table S3.** Common regions of gain /loss <10 Mb in size in classic lobular subtypes. **Table S4.** Regions of amplification occurring in more than one sample. **Table S5a.** Somatic mutations identified by whole exome sequencing in both the LCIS and ILC components in a single paired case. **Table S5b.** Somatic mutations identified by whole exome sequencing in ILC but not the LCIS component in a single paired case. **Table S5c.** Somatic mutations identified by whole exome sequencing in LCIS but not the ILC component in a single paired case. (DOCX 46 kb)
Additional file 2: Figure S1. Example of subclonal loss calculation using SNP array. **Figure S2.** Pure c-LCIS showing anueploidy and chromothripsis. **Figure S3.** Proportion of SCNA breakpoints in different subtypes of lobular cancer. (PDF 2813 kb)

## Abbreviations

cILC: classic invasive lobular cancer
cLCIS: classic lobular carcinoma *in situ*
cnLOH: copy number loss of heterozygosity
CNV: copy number variation
DCIS: ductal carcinoma *in situ*
ER: oestrogen receptor
FFPE: formalin-fixed paraffin-embedded tissue
FISH: fluorescence *in situ* hybridization
IDC: invasive ductal carcinoma
ILC: invasive lobular cancer
inv-cLCIS: classic lobular carcinoma *in situ* associated with ILC
LCIS: lobular carcinoma *in situ*
LOH: loss of heterozygosity
MIP: molecular inversion probe
pILC: pleomorphic invasive lobular cancer
pLCIS: pleomorphic lobular carcinoma *in situ*
SCNA: somatic copy number aberration
SNP: single nucleotide polymorphism
TAPS: Tumour Aberration Prediction Suite
TGCA: The Cancer Genome Atlas
WES: whole exome sequencing
