# Supplementary material for: PIK3CA mutations are common in lobular carcinoma in situ, but are not a biomarker of progression
Source: Breast Cancer Res. 2017 Jan 17;19:7. doi: 10.1186/s13058-016-0789-y (PMC5240238; doi:10.1186/s13058-016-0789-y)
Supplement: Additional file 1: Table S1a. — Clinico-pathological features of the discovery set - pure LCIS. Table S1b. Clinico-pathological features of the discovery set - inv-LCIS and ILC. Table S2a. Validation set: characteristics of the eight pure LCIS tumours that recurred. Table S2b. Validation set: characteristics of pure LCIS tumours that did not recur. Table S3. Common regions of gain /loss <10 Mb in size in classic lobular subtypes. Table S4. Regions of amplification occurring in more than one sample. Table S5a. Somatic mutations identified by whole exome sequencing in both the LCIS and ILC components in a single paired case. Table S5b. Somatic mutations identified by whole exome sequencing in ILC but not the LCIS component in a single paired case. Table S5c. Somatic mutations identified by whole exome sequencing in LCIS but not the ILC component in a single paired case. (DOCX 46 kb) [file 13058_2016_789_MOESM1_ESM.docx]

Table S1a: Clinico-pathological features of discovery set – pure LCIS

| **Pathology** | **Age at Diagnosis** | **Extensive LCIS*** | **Other Ipsilateral Pathology** | **Contralteral Pathology** | **LCIS –**  **How Diagnosed** |
| --- | --- | --- | --- | --- | --- |
| pure cLCIS | 60 |  | LG DCIS |  | mammogram |
| pure cLCIS | 59 |  |  |  | prophylactic mastectomy as strong FH of BC |
| pure cLCIS | 44 |  |  |  | incidental |
| pure cLCIS | 54 |  | ALH |  | mammogram |
| pure cLCIS | 58 |  | ADH |  | incidental |
| pure cLCIS | 42 |  | ALH |  | incidental |
| pure cLCIS | 60 | Yes | IG DCIS |  | Incidental |
| pure cLCIS | 50 | Yes | ADH |  | Mammogram |
| pure cLCIS | 51 | Yes |  | LCIS | Unknown |
| pure cLCIS | 52 |  | ALH |  | Incidental |
| pure cLCIS | 52 | Yes |  |  | Incidental |
| pure cLCIS | 49 |  | ALH |  | Unknown |
| pure cLCIS | 47 |  | ALH & ADH |  | Incidental |
| pure cLCIS | 58 |  |  |  | Incidental |
| pure cLCIS | 54 |  |  |  | Incidental |
| pure cLCIS | 52 |  |  |  | Incidental |
| pure cLCIS | 53 | Yes |  |  | Incidental |
| pure cLCIS | 52 | Yes |  |  | Incidental |
| pure cLCIS | 58 |  |  |  | Incidental |
| pure cLCIS | 50 |  |  |  | Incidental |
| pure cLCIS | 51 |  |  |  | Incidental |
| pure cLCIS | 50 |  | ALH |  | Incidental |
| pure cLCIS | 57 |  |  |  | Incidental |
| pure cLCIS | 43 |  |  | IG DCIS | Incidental |
| pure cLCIS | 51 |  | ADH |  | Incidental |
| pure cLCIS | 52 | Yes |  |  | Incidental |
| pure cLCIS | 50 | Yes | ALH |  | Incidental |

***> 10 acinar involved**

Table S1b: Clinico-pathological features of discovery set – invLCIS & ILC

| **Pathology** | **Age at Diagnosis** | **Extensive LCIS*** | **Other Ipsilateral Pathology** | **Contralateral Pathology** | **ILC ER status#** | **ILC Her2 status#** | **ILC grade** | **ILC nodal status** | **ILC multifocal** |
| --- | --- | --- | --- | --- | --- | --- | --- | --- | --- |
| cLCIS &ILC | 45 |  |  |  | positive | Negative | 2 | negative |  |
| cLCIS &ILC | 59 | Yes |  |  | positive | Unknown | 2 | positive |  |
| cLCIS &ILC | 58 |  | ALH |  | positive | Negative | 2 | negative |  |
| cLCIS &ILC | 51 |  |  |  | positive | Negative | 2 | positive |  |
| cLCIS &ILC | 41 |  |  |  | positive | Positive | 2 | positive |  |
| cLCIS &ILC | 39 |  | ADH |  | unknown | Unknown | 2 | positive |  |
| cLCIS &ILC | 57 |  |  |  | positive | Negative | 2 | negative |  |
| cLCIS &ILC | 52 |  |  |  | unknown | Unknown | 2 | positive |  |
| cLCIS &ILC | 51 |  |  |  | unknown | Unknown | 2 | negative |  |
| cLCIS &ILC | 60 | Yes |  | LCIS | positive | Negative | 3 | negative | Yes |
| cLCIS &ILC | 44 |  |  |  | positive | Negative | 3 | negative |  |
| cLCIS &ILC | 53 |  |  |  | positive | Negative | 2 | positive |  |
| cLCIS &ILC | 38 | Yes |  |  | positive | Negative | 2 | positive | Yes |
| cLCIS &ILC | 43 | Yes | ADH |  | positive | Negative | 2 | positive | Yes |
| cLCIS &ILC | 60 |  |  |  | positive | Negative | 2 | negative |  |
| cLCIS &ILC | 57 |  |  | ILC with ALH | positive | Negative | 2 | negative |  |
| cLCIS &ILC | 50 |  |  |  | positive | Negative | 2 | negative |  |
| cLCIS &ILC | 60 |  |  |  | positive | Unknown | 2 | negative |  |
| cLCIS &ILC | 53 |  |  |  | positive | Negative | 2 | positive |  |
| cLCIS &ILC | 46 | Yes |  |  | positive | Negative | 2 | negative | Yes |
| cLCIS &ILC | 50 | Yes |  |  | positive | Unknown | 2 | negative |  |
| cLCIS &ILC | 59 | Yes |  |  | positive | Negative | 2 | negative | Yes |
| cLCIS &ILC | 46 | Yes |  |  | positive | Negative | 2 | negative | Yes |
| cLCIS &ILC | 40 |  |  |  | positive | Negative | 2 | positive | Yes |
| cLCIS &ILC | 58 | Yes |  |  | positive | Negative | 2 | positive | Yes |
| cLCIS &ILC | 57 | Yes |  |  | positive | unknown | 2 | negative | Yes |
| cLCIS &ILC | 52 | Yes |  |  | positive | negative | 2 | negative |  |
| cLCIS &ILC | 56 |  | ALH |  | positive | unknown | 2 | unknown |  |

***> 10 acinar involved, # from path report**

Table S2a:

Validation Set: Characteristics of the eight pure LCIS cases that recurred

| **Age** | **Primary Diagnosis** | **Recurrent**  **Diagnosis** | **Time to recurrence** | **Side** | **Type of Surgery** | **CCND1**  **IHC**  **(proportion, Intensity)** | **CCND1**  **FISH** |
| --- | --- | --- | --- | --- | --- | --- | --- |
| 53 | cLCIS | Tubular, DCIS | 6yrs | Contralateral | WLE | H (5,3) | Normal |
| 55 | cLCIS | IDC, DCIS | 4yrs | Ipsilateral | EB | H (5,3) | Normal |
| 58 | cLCIS | pILC, cILC, pLCIS, | 2yrs | Contralateral | EB | H (5,3) | Gain |
| 46 | cLCIS | ILC , IDC | 10yrs | Ipsilateral | WLE | H (4,3) | Normal |
| 42 | cLCIS | LCIS, ILC | 3yrs | Ipsilateral | EB | H (4,3) | Normal |
| 48 | c&pLCIS | sILC, pILC, DCIS | 6yrs | Ipsilateral | WLE | H (5,3) | Amplified |
| 55 | cLCIS | IDC, DCIS | 2yrs | Contralateral | WLE | H (4,2) | Normal |
| 50 | ALH | ILC, LCIS, | 4yrs | Contralateral | WLE | I (3,2) | Normal |

Table S2b:

Validation Set: Characteristics of pure LCIS cases that did not recur

| **Age**  **At**  **Diagnosis** | **Primary Diagnosis** | **Follow up**  **(months)** | **Extensive**  **LCIS** | **Other**  **Ipsilateral Pathology** | **Type of**  **Surgery** | **Contra-**  **lateral**  **Pathology** | **Chemo-prevention** | **CCND1**  **IHC**  **(proportion, Intensity)** | **CCND1**  **FISH** |
| --- | --- | --- | --- | --- | --- | --- | --- | --- | --- |
| 54 | cLCIS | 76 |  |  | EB |  | No | I (2,1) | NP |
| 45 | cLCIS | 83 |  |  | EB |  | No | I (3,2) | NP |
| 41 | cLCIS | 333 |  |  | EB |  | No | H (5,3) | Normal |
| 35 | cLCIS | 293 |  |  | EB |  | No | N/L (1,1) | NP |
| 44 | cLCIS | 328 |  |  | EB |  | No | I (3,2) | NP |
| 55 | cLCIS | 204 |  | ALH | EB |  | No | N/L (0,0) | NP |
| 51 | cLCIS | 71 |  | IG DCIS | WLE |  | Unknown | N/L (0,0) | NP |
| 58 | cLCIS | 77 |  |  | WLE |  | No | H (5,2) | Normal |
| 36 | cLCIS | 60 |  |  | EB |  | No | I (1,3) | NP |
| 59 | cLCIS | 69 |  | IG-DCIS | WLE |  | Unknown | H (4,2) | NP |
| 59 | cLCIS | 67 |  | ALH | WLE |  | No | I (2,2) | NP |
| 51 | cLCIS | 60 |  | LG-DCIS | WLE |  | No | H (5,3) | Normal |
| 55 | cLCIS | 285 |  |  | EB |  | No | N/L (1,1) | NP |
| 51 | cLCIS | 98 |  |  | WLE |  | Unknown | I (1,3) | NP |
| 49 | cLCIS | 145 | Yes | ALH | WLE |  | No | I (2,3) | NP |
| 56 | cLCIS | 96 | Yes |  | Reduction | DCIS &LCIS | No | H (5,3) | Normal |
| 37 | cLCIS | 66 |  | ALH & DCIS | WLE |  | No | H (5,2) | Normal |
| 49 | cLCIS | 104 | Yes |  | WLE |  | No | H (5,3) | Amplified |
| 50 | cLCIS | 224 |  |  | EB |  | Unknown | N/L (0,0) | NP |
| 56 | cLCIS | 70 |  |  | EB |  | No | H (5,3) | NP |
| 50 | pLCIS | 63 | Yes | ADH | WLE |  | Unknown | N/L (1,1) | NP |
| 54 | cLCIS | 74 |  | ADH | WLE |  | No | I (2,1) | NP |
| 59 | cLCIS | 85 | No | ALH & ADH | WLE |  | No | I (2,1) | NP |
| 51 | cLCIS | 85 | Yes |  | WLE | LCIS & LG DCIS | No | I (2,1) | NP |

Table S3:

Common regions of gain /loss <10Mb in size in Classical Lobular Subtypes

| **Chro** | **Region** | **Genes** | **Pure-cLCIS** | | | **Inv-cLCIS** | | | **Paired ILC** | | |
| --- | --- | --- | --- | --- | --- | --- | --- | --- | --- | --- | --- |
|  |  |  | **Gain** | **Cn-LOH** | **Loss** | **Gain** | **Cn-LOH** | **Loss** | **Gain** | **Cn-LOH** | **Loss** |
| 1 | 46201546-  46594140 | MAST2,  PIK3R3 | 6 | 0 | 0 | 7 | 0 | 0 | 0 | 0 | 3 |
| 3 | 69787304-  69941100 | MITF | 8 | 1 | 0 | 8 | 1 | 1 | 7 | 0 | 1 |
| 9 | 128165696-  128481324 | MAPKAP1 | 5 | 3 | 1 | 8 | 1 | 0 | 4 | 0 | 0 |
| 17 | 29417402-  29721675 | NF1, OMG,  EVI2A | 6 | 0 | 1 | 6 | 1 | 1 | 0 | 0 | 3 |
| 17 | \| 4796928-  13070380 \|  \| \| --- \| --- \| | TP53…  AURKB…  PIK3R5…  GAS7…  MAP2K4 | 0 | 0 | 7 | 0 | 1 | 8 | 0 | 0 | 12 |

Table S4:

Regions of amplification occurring in more than one sample

| **Chrom** | **Region** | **Genes** | **N^o^ of cases** | | |
| --- | --- | --- | --- | --- | --- |
|  |  |  | **Pure-cLCIS** | **Inv-cLCIS** | **cILC** |
| 1 | 152033587-  209766930 | TCHHL1…APOBEC4…  MDM4…TMEM183A | 2 | 0 | 1 |
| 1 | 243613003-  244164912 | SDCCAG8, AKT3 | 4 | 4 | 4 |
| 1 | 244207377-  249250621 | ZBTB18…OR2T34…  PGBD2 | 2 | 0 | 1 |
| 3 | 69787304- 69941100 | MITF | 2 | 1 | 1 |
| 5 | 80719283-  81481720 | SSBP2, ATG10 | 0 | 1 | 1 |
| 6 | 168339982-  168569157 | MLLT4, KIF25, FRMD1 | 1 | 0 | 3 |
| 9 | 128010996-  129653879 | GAPVD1, MAPKAP1, PBX3, MVB12B, LMX1B, ZBTB43, ZBTB34 | 1 | 1 | 0 |
| 10 | 89481311-  89527885 | PAPSS2, ATAD1 | 1 | 0 | 1 |
| 11 | 48177965-  49926883 | PTPRJ, OR4B1, OR4X2, OR4X1, OR4S1, OR4C3, OR4C5, OR4A47, TRIM49B, TRIM64C, FOLH1 | 1 | 0 | 1 |
| 11 | 69409496-  69635309 | CCND1, ORAOV1, FGF19, FGF4, FGF3 | 0 | 2 | 5 |
| 12 | 20972343-  29910025 | SLCO1B3….KRAS….  TMTC1 | 1 | 0 | 1 |
| 12 | 69807990-  70319728 | FRS2, CCT2, LRRC10, BEST3, RAB3IP, MYRFL | 0 | 2 | 1 |
| 14 | 101340331-  101537115 | RTL1, SNORD, MIR | 1 | 2* | 0 |
| 16 | 11719144-  12670903 | LITAF, SNN, TXNDC11, ZC3H7A, RSL1D1, GSPT1,TNFRSF17, SNX29 | 2 | 1 | 2 |
| 17 | 17030102-  21592626 | SLC47A2....TOP3A ....  MAP2K3……MAPK7 ...... TOM1L2........ FLII | 0 | 1 | 1 |
| 18 | 731781-  831538 | YES1 | 2 | 0 | 0 |

*these two samples did not have paired ILC

Table S5a:

Somatic mutations identified by whole exome sequencing in both the LCIS and ILC components in a single paired case

| **Chrom** | **Gene** | **Mutation** | **Type** |
| --- | --- | --- | --- |
| chr1 | HSPG2 | c.G5071A | nonsynonymous SNV |
| chr1 | ROR1 | c.C719G | nonsynonymous SNV |
| chr3 | PIK3CA | c.G1633A | nonsynonymous SNV |
| chr3 | PIK3CA | c.G2176A | nonsynonymous SNV |
| chr5 | DOCK2 | c.C4786T | stopgain SNV |
| chr10 | COX15 | c.1128_1131del | frameshift deletion |
| chr11 | OR56B1 | c.A367G | nonsynonymous SNV |
| chr13 | PROSER1 | c.C1507G | nonsynonymous SNV |
| chr16 | CDH1 | c.1231_1232insG | frameshift insertion |
| chr17 | PTRF | c.C132G | nonsynonymous SNV |
| chrX | ATP11C | c.C2812G | nonsynonymous SNV |
| chrX | ATRX | c.T3076G | nonsynonymous SNV |

Table S5b:

Somatic mutations identified by whole exome sequencing in ILC but not the LCIS component in a single paired case

| **Chrom** | **Gene** | **Mutation** | **Type** |
| --- | --- | --- | --- |
| chr1 | SLC45A1 | c.G2305A | nonsynonymous SNV |
| chr1 | LEPR | c.C384A:p.N128K | nonsynonymous SNV |
| chr12 | AGAP2 | c.C1829A | nonsynonymous SNV |
| chr13 | RB1 |  | splicing |
| chr14 | ARID4A | c.A1856T | nonsynonymous SNV |
| chr14 | SIX1 |  | splicing |
| chr14 | AK7 | c.1005_1007del | nonframeshift deletion |
| chr14 | NUBPL | c.C79G | nonsynonymous SNV |
| chr16 | PTX4 | c.G229A | nonsynonymous SNV |
| chr17 | GLTPD2 | c.T322G | nonsynonymous SNV |
| chr17 | MAP2K4 |  | splicing |
| chr18 | ATP8B1 |  | splicing |
| chr19 | BABAM1 | c.T217G | nonsynonymous SNV |
| chr20 | ZNF341 | c.G2512A | nonsynonymous SNV |
| chr3 | VGLL3 | c.T17G | nonsynonymous SNV |
| chr4 | PRDM5 | c.T28G | nonsynonymous SNV |
| chr5 | HAND1 | c.T214C | nonsynonymous SNV |
| chr7 | NCAPG2 |  | splicing |
| chrX | UBE2A | c.G88C | nonsynonymous SNV |
| chrX | PDHA1 |  | intronic |
| chrX | CXorf22 |  | splicing |
| chrX | SMS | c.G578A | nonsynonymous SNV |
| chrX | DCAF12L1 | c.C236T | nonsynonymous SNV |

Table S5c:

Somatic mutations identified by whole exome sequencing in LCIS but not the ILC component in a single paired case

| **Chrom** | **Gene** | **Mutation** | | **Type** | |  |  |
| --- | --- | --- | --- | --- | --- | --- | --- |
| chr1 | NADK | c.A724G |  | nonsynonymous SNV | | |  |
| chr1 | OBSCN |  |  | splicing | | |  |
| chr12 | NACA |  |  | intronic | | |  |
| chr17 | ABCC3 | c.A1681C |  | nonsynonymous SNV | | |  |
| chr17 | LGALS9B | c.G1039A |  | nonsynonymous SNV | | |  |
| chr19 | ACTN4 | c.G1234C |  | nonsynonymous SNV | | |  |
| chr6 | GGNBP1,LINC00336 |  |  | ncRNA | | |  |
| chr8 | DLGAP2 | c.C933A |  | nonsynonymous SNV | | |  |
| chr9 | ASTN2 | c.T2423G |  | nonsynonymous SNV | | |  |
| chrX | ARX | c.C956G |  | | nonsynonymous SNV | | |
| chrX | KIF4A | c.G3688C |  | | nonsynonymous SNV | | |
