# Supplementary material for: PIK3CA mutations are common in lobular carcinoma in situ, but are not a biomarker of progression
Source: Breast Cancer Res. 2017 Jan 17;19:7. doi: 10.1186/s13058-016-0789-y (PMC5240238; doi:10.1186/s13058-016-0789-y)

Figure S1: Example of subclonal loss calculation

(a) Log ratio and BAF plot of inv-cLCIS derived from TAPS analysis, showing a subclonal loss on chromosome 3 and a clonal loss on chromosome 6. Chromosome 4 and 5 harbour long stretches of diploid 2m1 segments.

(b) An allelic imbalance versus average log ratio plot. Chromosomal segments with absolute copy number of 1m0 in 100% of tumour cells (clonal loss), of 1m0 as a subclonal event, of 2m1 are shown as brown, red and purple dots, respectively. To estimate clonal loss present in 100% of the tumour, we first took the centroids of BAF and logR values from the 1m0 segments on chromosome 6. We then established the centroid of all diploid segments and defined the Euclidean distance between clonal loss centroid and diploid state centroid, indicated by the black line. Then we calculated the Euclidean distance between subclonal loss centroid and diploid state centroid, indicated by the red line. The subclonality of these segments were defined as proportion of the later Euclidean distance to the clonal loss defined Euclidean distance.

A

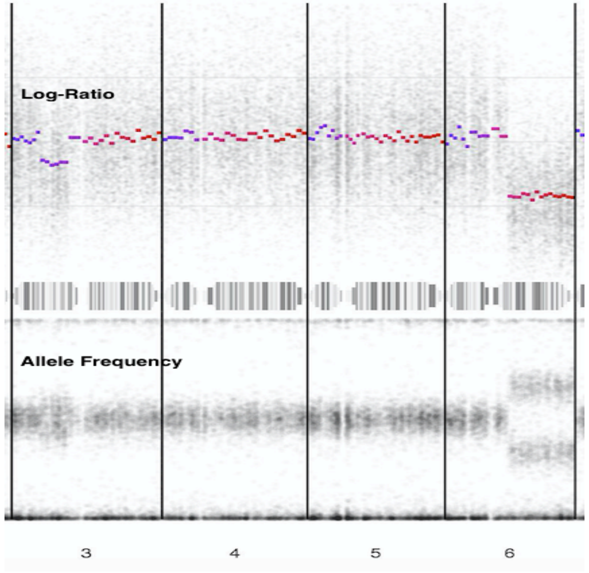

B

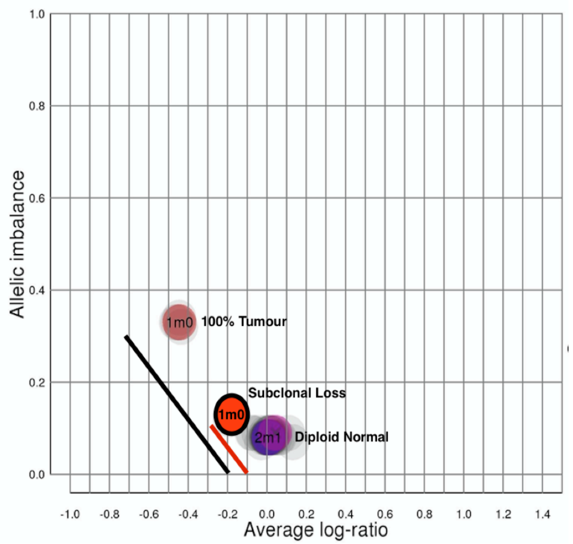

Figure S2: Pure c-LCIS showing: anueploidy and chromothripsis

Anueploid Sample

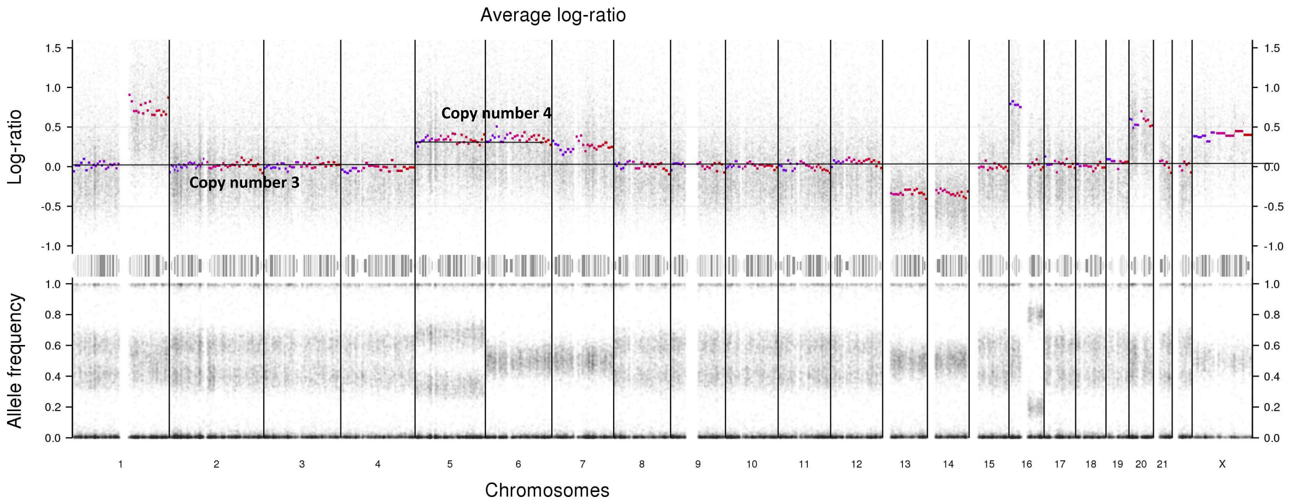

Chr6: Chromothripsis

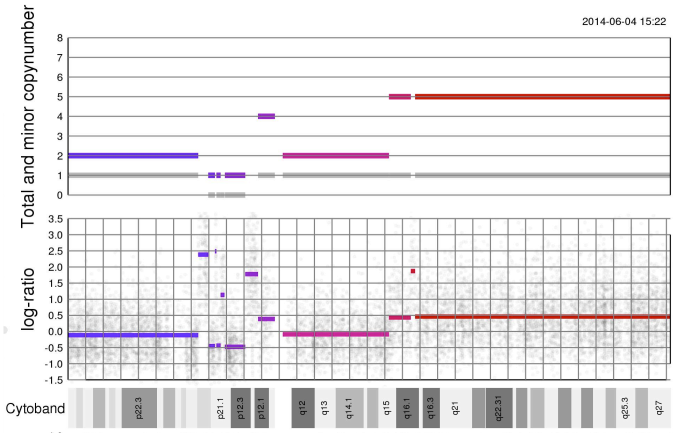

Chr17: Chromothripsis

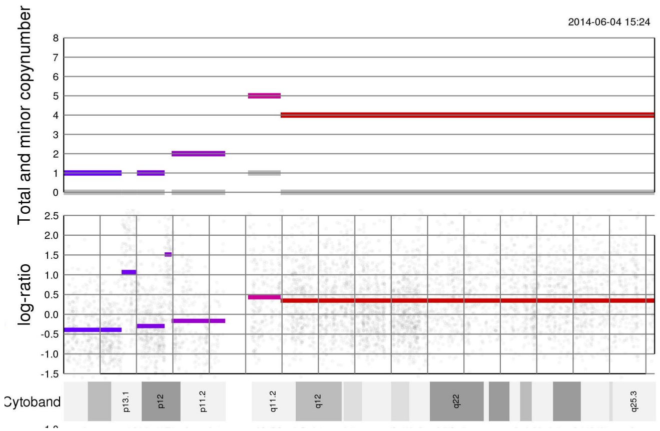

Figure S3: Proportion of SCNA breakpoints in different subtypes of classical lobular cancer

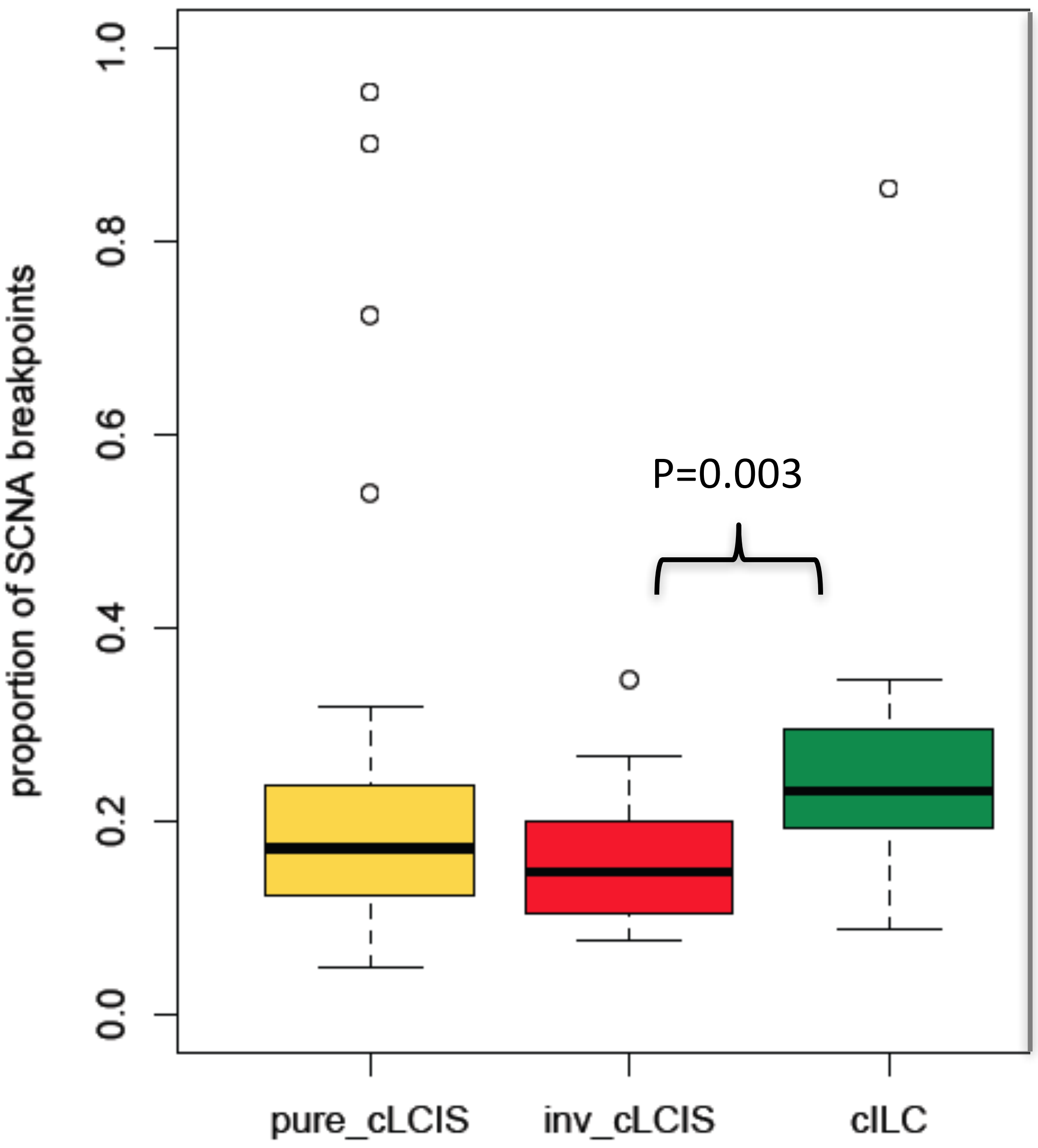

Supplement: Additional file 2: Figure S1. — Example of subclonal loss calculation using SNP array. Figure S2. Pure c-LCIS showing anueploidy and chromothripsis. Figure S3. Proportion of SCNA breakpoints in different subtypes of lobular cancer. (PDF 2813 kb) [file 13058_2016_789_MOESM2_ESM.pdf]
